# A Novel Predictive Model for Adrenocortical Carcinoma Based on Hypoxia- and Ferroptosis-Related Gene Expression

**DOI:** 10.3389/fmed.2022.856606

**Published:** 2022-05-16

**Authors:** Tianyue Zhang, Xiaoxiao Song, Jie Qiao, Ruiliang Zhu, Yuezhong Ren, Peng-Fei Shan

**Affiliations:** Department of Endocrinology, The Second Affiliated Hospital of Zhejiang University School of Medicine, Hangzhou, China

**Keywords:** adrenocortical carcinoma, hypoxia, ferroptosis, predictive model, gene expression

## Abstract

**Background:**

The impact of hypoxia on ferroptosis is important in cancer proliferation, but no predictive model combining hypoxia and ferroptosis for adrenocortical carcinoma (ACC) has been reported. The purpose of this study was to construct a predictive model based on hypoxia- and ferroptosis-related gene expression in ACC.

**Methods:**

We assessed hypoxia- and ferroptosis-related gene expression using data from 79 patients with ACC in The Cancer Genome Atlas (TCGA). Then, a predictive model was constructed to stratify patient survival using least absolute contraction and selection operation regression. Gene expression profiles of patients with ACC in the Gene Expression Omnibus (GEO) database were used to verify the predictive model.

**Results:**

Based on hypoxia-related gene expression, 79 patients with ACC in the TCGA database were divided into three molecular subtypes (C1, C2, and C3) with different clinical outcomes. Patients with the C3 subtype had the shortest survival. Ferroptosis-related genes exhibited distinct expression patterns in the three subtypes. A predictive model combining hypoxia- and ferroptosis-related gene expression was constructed. A nomogram was constructed using age, sex, tumor stage, and the predictive gene model. Gene ontology and Kyoto Encyclopedia of Genes and Genomes analyses revealed that the gene signature was mainly related to the cell cycle and organelle fission.

**Conclusion:**

This hypoxia-and ferroptosis-related gene signature displayed excellent predictive performance for ACC and could serve as an emerging source of novel therapeutic targets in ACC.

## Introduction

Adrenocortical carcinoma (ACC) is a rare, aggressive, heterogeneous malignancy derived from the cortex of the adrenal gland. The 5-year survival rate of ACC ranges from 0 to 28% (1–3). Despite the marked variation in survival, prognostic factors have not been definitively investigated. Although the age at diagnosis, tumor characteristics, tumor stage, and cortisol production is believed to be adverse prognostic factors, there are few reliable biomarkers to aid clinical assessment (4–6). Therefore, discovering effective biomarkers is essential for improving ACC prognosis.

Hypoxia is a common feature of solid tumors because of their rapid proliferation and abnormal vascularization. Studies have found that hypoxia-inducible factor 1 (HIF-1) signaling is associated with metastasis, immune evasion, resistance to therapy, and increased mortality in cancer (7, 8). However, the effects of HIF-1 activity in ACC are unclear (7, 9). Recently, a bioinformatic study found that a hypoxia-related gene signature could predict prognosis and reflect the immune microenvironment in ACC (10). Further, increasing evidence indicates that the organic response to tumor hypoxia includes alteration of ferroptosis (9, 11). Ferroptosis is an iron-dependent form of non-apoptotic cancer cell death (12), and ferroptosis-related genes are closely linked to the prognosis of various cancers, including ACC (13–19). Although the impact of hypoxia on ferroptosis is important in cancer proliferation, no predictive model combining hypoxia and ferroptosis for ACC has been reported. Hence, a model based on hypoxia- and ferroptosis-related gene expression might be useful for predicting prognosis in ACC patients.

This study analyzed the relationship between hypoxia-related molecular subtypes and ferroptosis-related gene expression and constructed a reliable predictive model for ACC based on hypoxia- and ferroptosis-related gene expression.

## Materials and Methods

### Patients and Datasets

**Figure 1:**
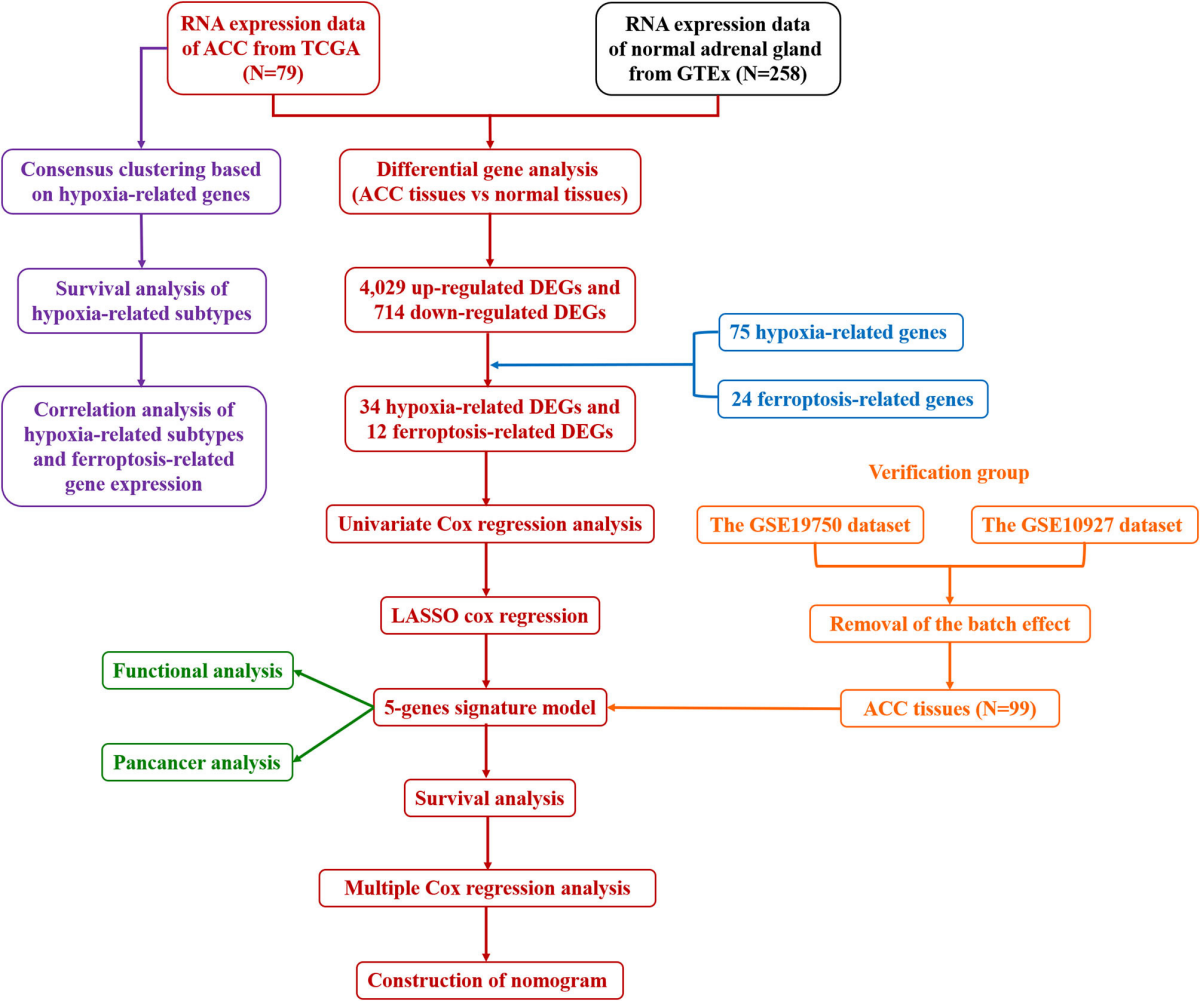
The detailed flow chart of this study. ACC, adrenocortical carcinoma; TCGA, The Cancer Genome Atlas; GTEx, Genotype-Tissue Expression; DEGs, differentially expressed genes; LASSO, least absolute shrinkage and selection operation.

The gene expression data and clinical information of 79 patients with ACC were obtained from The Cancer Genome Atlas (TCGA) on June 13, 2021 (https://portal.gdc.cancer.gov/repository). The gene expression data of 258 normal adrenal gland samples were obtained from the Genotype-Tissue Expression (GTEx) database (https://toil.xenahubs.net/download/gtex_RSEM_gene_tpm.gz). The GSE19750 and GSE10927 datasets from the Gene Expression Omnibus (GEO) database were used as validation cohorts (https://www.ncbi.nlm.nih.gov/geo/). The “removeBatchEffect” function of the R package “limma” (version 4.1.0) was used to remove the batch effects of GSE19750 and GSE10927. The detailed flow chart of our study was displayed in Figure 1.

### Consensus Clustering

Seventy-nine patients with ACC were clustered into three groups based on hypoxia-related gene expression profiles using the R software package “ConsensusClusterPlus” (version 1.54.0). The maximum number of clusters is 6, and 80% of the total sample is drawn 100 times, clusterAlg = “hc,” innerLinkage = “ward.D2.” Clustering heatmaps were created using the R software package “pheatmap” (version 1.0.12). The Kaplan–Meier survival analysis was then used to compare the survival difference among three groups using the R software package “survival” (version 3.2-13) and “survminer” (version 0.4.9). The expression distribution of ferroptosis-related genes in three hypoxia-related subtypes was analyzed by the Wilcoxon test. The box plot was implemented by the R software package “ggplot2.” Ferroptosis-related gene expression heat map was implemented by the R software package “pheatmap.”

### Differential Gene Analysis

The R software package “Limma” (version 4.1.0) was used for differential gene analysis by comparing the gene expression pattern in ACC tissues to that in normal tissues. The adjusted *P*-value was analyzed to correct for false positive results in TCGA or GTEx. “Adjusted *P* < 0.05 and Log (Fold Change) >1 or Log (Fold Change) < −1” were defined as the thresholds for identifying the differentially expressed genes (DEGs). The volcano plot of DEGs was implemented by the R software package “ggplot2.” The hierarchical clustering heat map was implemented by the R software package “pheatmap.”

### Univariate Cox Regression Analysis

Seventy-five hypoxia-related genes (Supplementary Table S1) and 24 ferroptosis-related genes (Supplementary Table S2) were derived from published literature (17, 20). Subsequently, 34 hypoxia-related DEGs and 12 ferroptosis-related DEGs were extracted. For Kaplan–Meier curves, *P* values and hazard ratio (HR) with 95% confidence interval (CI) were generated by log-rank tests and univariate Cox proportional hazards regression using the R software package “survival” (version 3.2-13) and “survminer” (version 0.4.9). Prognosis-related genes with P < 0.05 were screened for the establishment of a hypoxia and ferroptosis-related gene signature.

### Protein–Protein Interaction Network

A protein–protein interaction (PPI) network was constructed using the web tool called “STRING” (https://string-db.org/) to explore the interactions among the above key genes that affected overall survival (OS).

### Establishment and Validation of a Hypoxia- and Ferroptosis-Related Gene Signature

The key genes that affected OS were further screened with the least absolute shrinkage and selection operation (LASSO) Cox regression model, using the R software package “glmnet” (version 4.1-1). The risk score was calculated by combining the regression coefficients and the corresponding gene expression levels. Based on the median risk score, patients with ACC were divided into high- and low-risk groups. The Kaplan–Meier survival curves were generated using the R software package “survival” (version 3.2-13) and “survminer” (version 0.4.9). Receiver operator characteristic (ROC) curve analysis was performed to test the sensitivity and specificity of the predictive model using the R software package “timeROC” (version 0.4). Multivariate Cox regression analysis was performed to evaluate whether the risk score was an independent prognostic factor for OS. Age, gender, stage, and the risk score were used to construct a nomogram. The GSE19750 and the GSE10927 dataset were utilized as the validation cohort. The Kaplan–Meier survival analysis and the ROC curve analysis were repeated in the same way as before.

### Tumor Mutation Burden

Tumor mutation burden (TMB) is the total amount of substitutions, insertions, or deletions per mega bases in the exon-coding regions in tumors and is a potential biomarker for predicting response to immune checkpoint inhibitor therapy (21). The expression distribution of 5 hub genes in ACC and normal adrenal gland tissues was analyzed by the student's *t*-test. The Sankey diagram was drawn with each column representing a characteristic variable, different colors representing different types, and lines representing the distribution of the same sample in different characteristic variables. Then, Spearman's correlation analysis was used to explore the correlation of the gene expression and TMB.

### Functional Enrichment Analysis

Based on the median risk score, patients with ACC were divided into high- and low-risk groups. Differential gene analysis was performed between the two groups. Gene Ontology (GO) and Kyoto Encyclopedia of Genes and Genomes (KEGG) pathway analyses were conducted using the R software package “clusterProfiler” (version 4.0.5). The R software package “GSVA” was used to analyze the correlation between genes and pathways (version 1.42.0).

### Pan-Cancer Analysis

The gene expression data of 33 kinds of tumors were obtained from the TCGA database. Spearman's correlation analysis was used to explore the correlation of the gene expression and TMB in 33 kinds of tumors.

### Statistical Analysis

Statistical analyses were conducted through R language (version 4.0.3). Differences between the two groups were analyzed via the student's *t*-test. Differences among the three groups were analyzed via the Wilcoxon test. The statistical methods related to bioinformatic analysis are detailed in the corresponding sections. *P* < 0.05 was considered statistically significant.

## Results

### Three Molecular Subtypes of Hypoxia-Related Gene Expression in ACC

**Figure 2:**
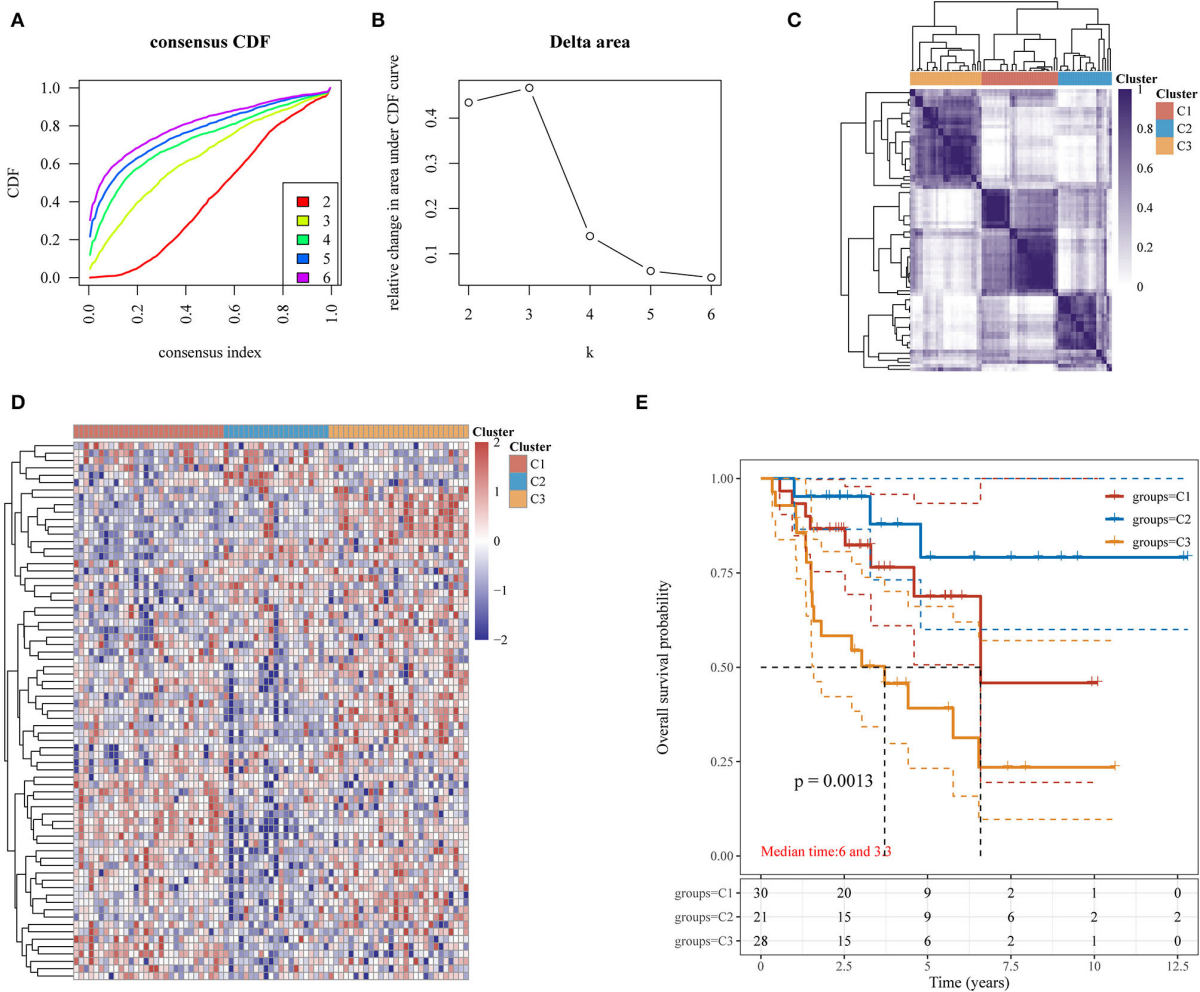
Identification of three molecular subtypes based on hypoxia-related gene expression in ACC. **(A)** Consensus CDF; **(B)** Delta area; **(C)** Heatmap of consensus clustering solution (*k* = 3) for hypoxia-related genes in ACC (*n* = 79); **(D)** Heatmap for hypoxia-related gene expression among the three hypoxia-related subtypes; **(E)** Kaplan-Meier OS curves for patients with ACC among the three hypoxia-related subtypes. ACC, adrenocortical carcinoma; CDF, cumulative distribution function; OS, overall survival.

The clinicopathologic features of 79 patients in the TCGA-ACC cohort are shown in Table 1. Our study classified these 79 ACC samples into molecular subtypes based on the expression profiles of hypoxia-related genes using the ConsensusClusterPlus package. The classification was reliable and stable at *k* = 3; therefore, the samples were divided into C1, C2, and C3 subtypes (Figures 2A–D). Patients with the C3 subtype had shorter survivals than those with the C1 and C2 subtypes (*p* = 0.0013; Figure 2E). Therefore, hypoxia-related gene expression can be used to characterize three ACC subtypes with distinct clinical outcomes.

**Table 1:** The clinicopathologic features of 79 patients in the TCGA-ACC cohort.

**Characteristics**	***N* = 79(%)**
Gender
Male	31 (39.24)
Female	48 (60.76)
Age (year)
≤ 60	61 (77.22)
>60	18 (22.78)
T stage
T1	9 (11.69)
T2	42 (54.54)
T3	8 (10.39)
T4	18 (23.38)
N stage
N0	68 (88.31)
N1	9 (11.69)
M stage
M0	62 (80.52)
M1	15 (19.48)
TNM stage
I	9 (11.69)
II	37 (48.05)
III	16 (20.78)
IV	15 (19.48)

*TCGA, The Cancer Genome Atlas; ACC, adrenocortical carcinoma; TNM stage, Tumor Node Metastasis stage*.

### Relationship Between the Three Hypoxia-Related Subtypes and Expression of Ferroptosis-Related Genes

**Figure 3:**
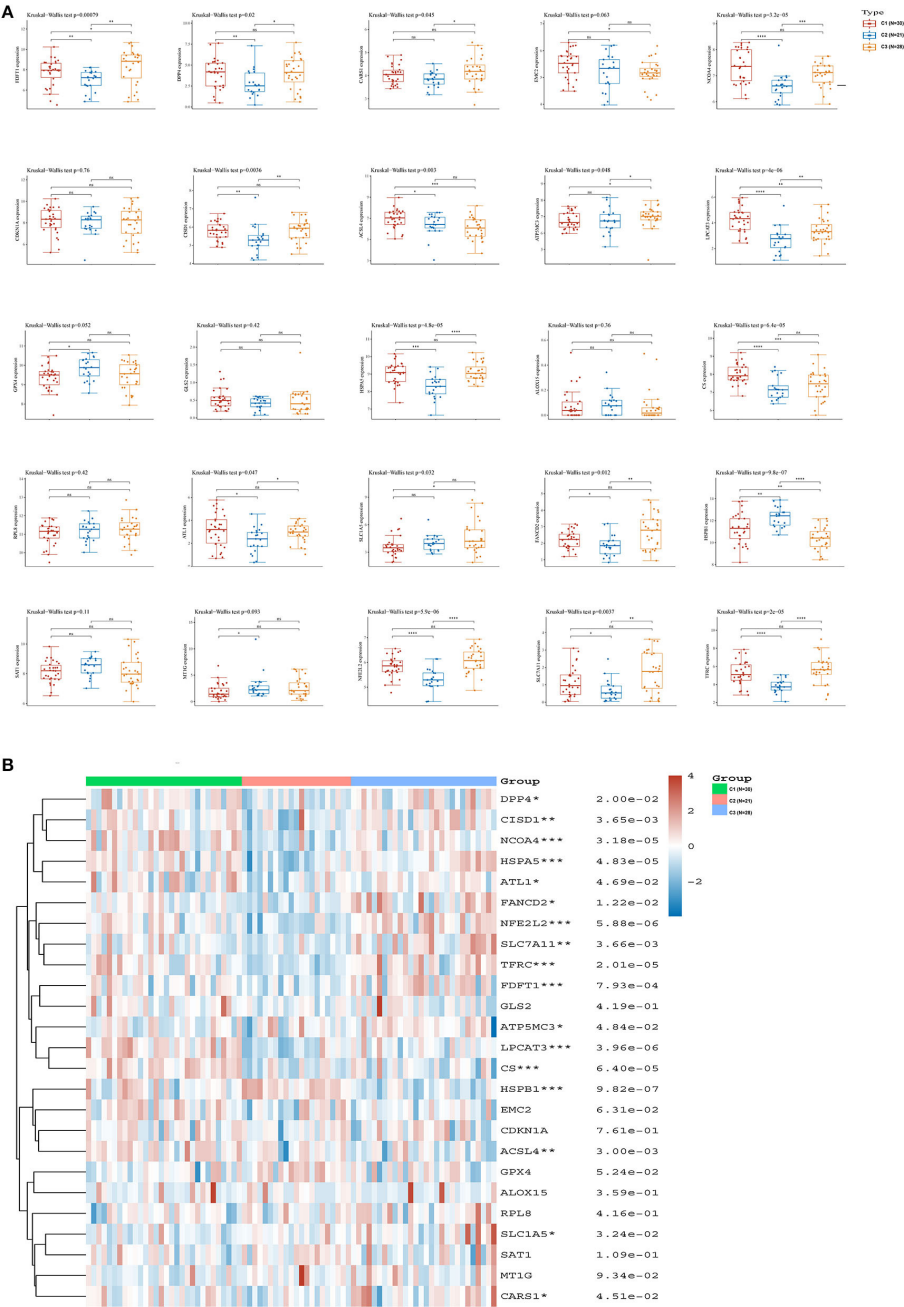
Relationship between hypoxia-related subtypes and ferroptosis in ACC. **(A)** The expression distribution of ferroptosis-related genes among the three hypoxia-related subtypes; **(B)** Ferroptosis-related gene expression heatmap. ACC, adrenocortical carcinoma. *: *P* < 0.05; **: *P* < 0.01; ***: *P* < 0.001.

We analyzed the associations between the three hypoxia-related subtypes and the expression of ferroptosis-related genes in ACC. We found that 17 of 25 ferroptosis related genes, including acyl-CoA synthetase long-chain family member 4 (ACSL4), atlastin GTPase 1 (ATL1), ATP synthase membrane subunit C locus 3 (ATP5MC3), cysteinyl tRNA synthetase 1 (CARS1), CDGSH iron sulfur domain 1 (CISD1), citrate synthase (CS), dipeptidyl-dipeptidase-4 (DPP4), Fanconi anemia complementation group D2 (FANCD2), farnesyl-diphosphate farnesyltransferase 1 (FDFT1), heat shock protein family A member 5 (HSPA5), heat shock protein beta 1 (HSPB1), lysophosphatidylcholine acyltransferase 3 (LPCAT3), nuclear receptor coactivator 4 (NCOA4), nuclear factor, erythroid 2 like 2 (NFE2L2), solute carrier family 1 member 5 (SLC1A5), solute carrier family 7 member 11 (SLC7A11), and transferrin receptor (TFRC), were significantly differentially expressed in the three hypoxia-related subtypes (Figure 3A). Among these ferroptosis-related genes, the expressions of FDFT1 and ATP5MC3 were significantly higher in C3 samples than in C1 and C2 samples. By contrast, the expression of HSPB1 was significantly lower in C3 samples than in C1 and C2 samples. The heatmap showed similar results (Figure 3B). Therefore, these three hypoxia-related subtypes had a strong association with ferroptosis in ACC.

### Establishment of a Predictive Model Based on Hypoxia- and Ferroptosis-Related Gene Expression

**Figure 4:**
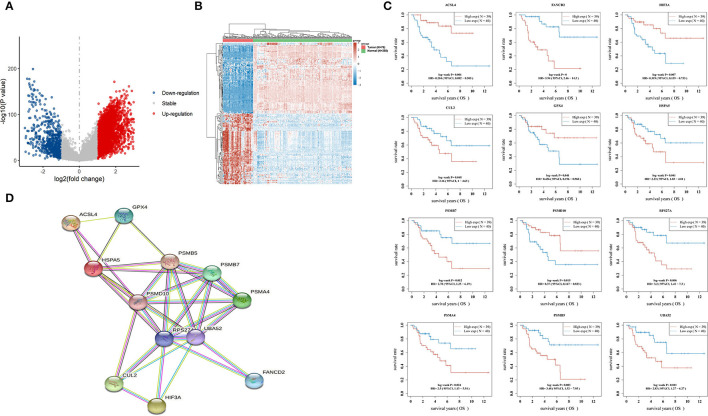
Hypoxia- and ferroptosis-related key genes in ACC. **(A)** Volcano plots indicating up-expressed and down-expressed genes in ACC compared with normal tissues; **(B)** Hierarchical clustering analysis of genes between ACC and normal tissues; **(C)** 12 genes significantly correlated with OS time of patient with ACC; **(D)** A PPI network of 12 genes retrieved from String. ACC, adrenocortical carcinoma; OS, overall survival; PPI, protein-protein interaction; String: https://string-db.org/.

**Figure 5:**
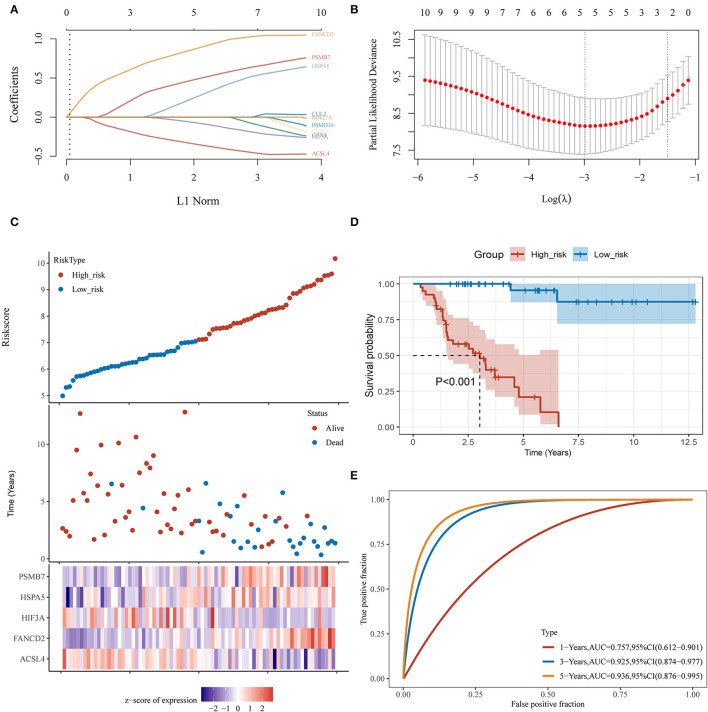
Establishment of a hypoxia- and ferroptosis-related predictive model. **(A)** LASSO coefficient profiles of 12 hypoxia-related and ferroptosis-related genes; **(B)** LASSO regression with 10-fold cross-verification; **(C)** Heatmap of the expression profiles of 5 prognostic genes in low-risk and high-risk group; **(D)** Kaplan-Meier survival analysis of the gene signature; **(E)** Time-dependent ROC analysis of the gene signature. ACC, adrenocortical carcinoma; LASSO, least absolute shrinkage and selection operation; ROC, receiver operating characteristic curve.

**Figure 6:**
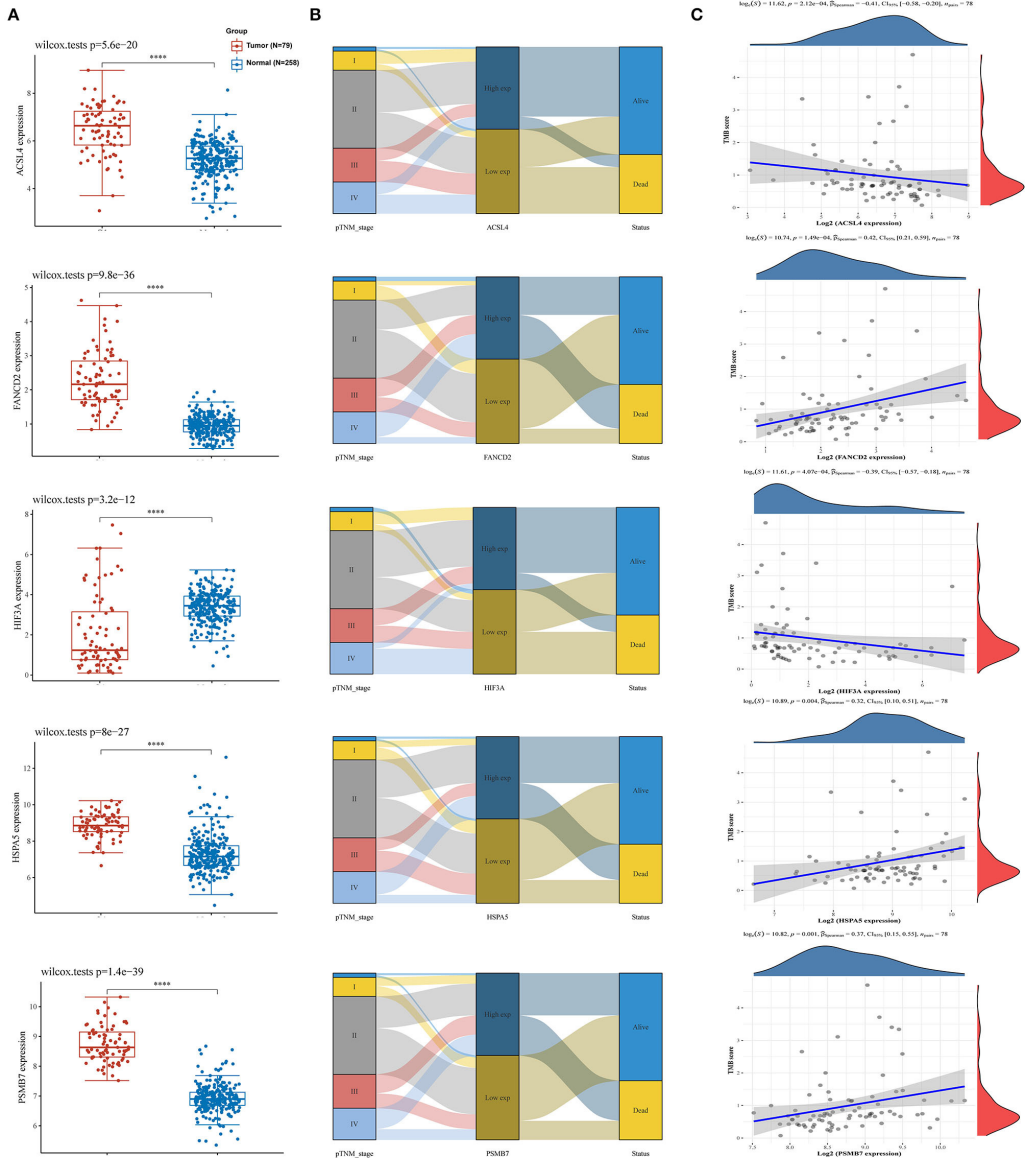
The interaction of five hub genes in the predictive model and TMB. **(A)** The expression distribution of 5 genes in ACC and normal tissues; **(B)** Sankey diagram of pTNM stage, gene expression and survival status; **(C)** Correlation analysis of gene expression and TMB. TMB, tumor mutation burden; ACC, adrenocortical carcinoma; pTNM stage, pathology Tumor Node Metastasis stage. *: *P* < 0.05; **: *P* < 0.01; ***: *P* < 0.001.

Using the limma package for analysis of differential gene expression in 79 ACC samples and 258 normal adrenal gland samples (Adjusted *P* < 0.05; |log2FoldChange| > 1), 4,743 DEGs were obtained, including 4,029 upregulated and 714 downregulated genes (Figures 4A,B). Next, 34 hypoxia-related DEGs and 12 ferroptosis-related DEGs were separately extracted from 75 previously identified hypoxia-related genes and 24 previously identified ferroptosis-related genes. After further screening these key genes by log-rank tests and univariate Cox proportional hazards regression, we found that 12 genes were significantly correlated with OS in patients with ACC (Figure 4C). A PPI network of 12 genes was then retrieved from String to explore the interaction among these genes (Figure 4D). We then constructed a LASSO Cox regression model (Figures 5A,B). The risk score was calculated based on the results of the LASSO Cox regression model, as follows: (−0.3843) ^*^ ACSL4 + (0.9418) ^*^ FANCD2 + (−0.1097) ^*^ HIF3A + (0.3538) ^*^ HSPA5 + (0.5395) ^*^ PSMB7 (Figure 5C). Seventy-nine patients with ACC were then separated into high- and low-risk groups based on the median risk score. Our results showed that patients in the high-risk group exhibited shorter OS than those in the low-risk group (*P* < 0.001; Figure 5D). We further verified the predictive efficacy of this model using ROC curve analysis. The AUC of the model for predicting OS at 3 years was 0.925 (Figure 5E), suggesting that the genetic model had accurate and robust prognostic prediction performance. To further explored the five hub genes (ACSL4, FANCD2, HIF3A, HSPA5, and PSMB7), we found that the expression of five hub genes in ACC was significantly different from that in the normal tissues (Figure 6A). Figure 6B represented the relationship among the expression of hub genes, the tumor stages, and survival status. We further found that each gene was significantly associated with TMB (Figure 6C).

### Evaluation of the Reliability and Stability of the Predictive Model

**Figure 7:**
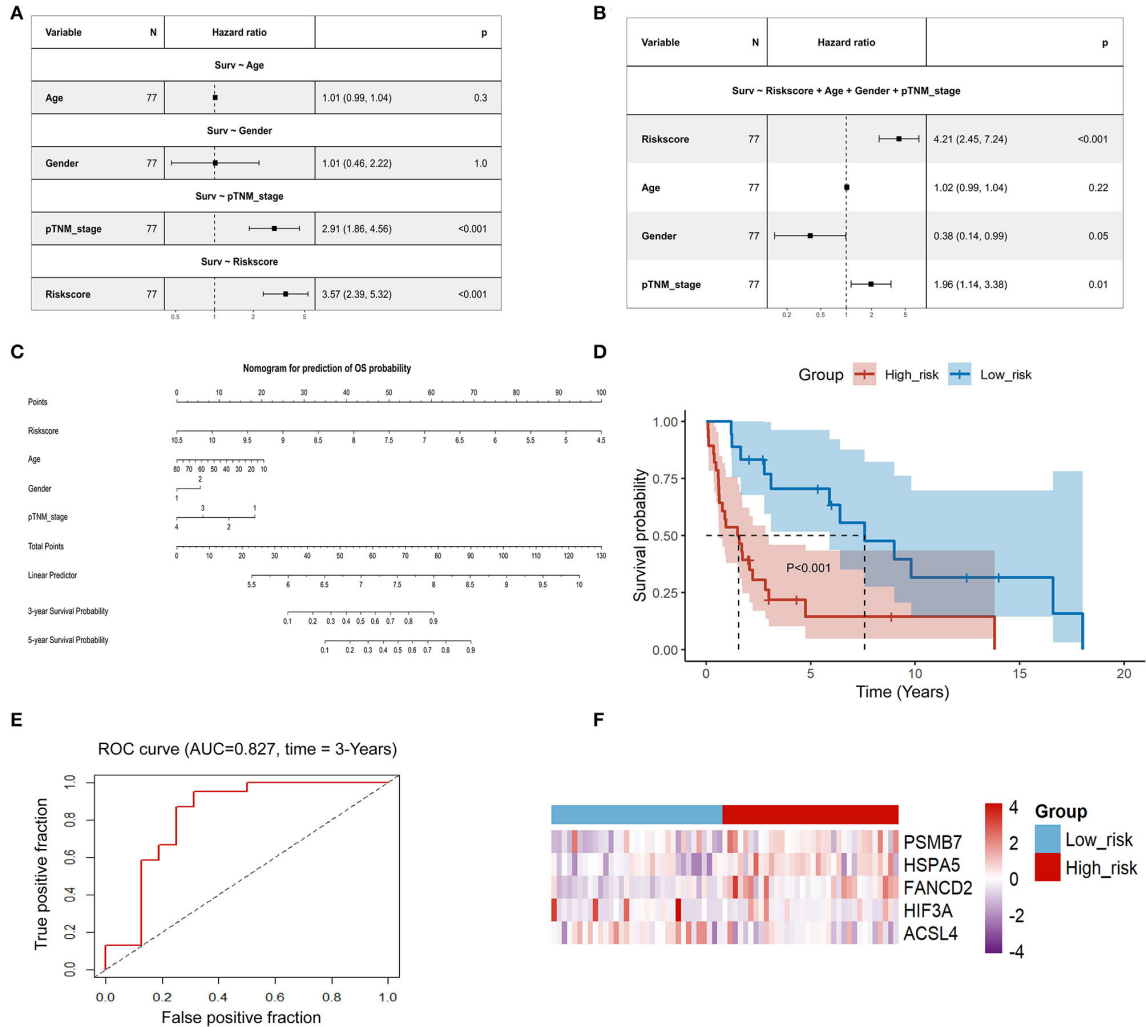
Evaluation of the reliability and the stability of the predictive model in ACC. **(A)** Univariate Cox regression analysis of age, gender, stage, and risk score in the TCGA-ACC set; **(B)** Multivariate Cox regression analysis of age, gender, stage, and risk score in the TCGA-ACC set; **(C)** A nomogram for prediction of OS probability; **(D)** Kaplan-Meier analysis of the gene signature in pooled data from the GSE19750 dataset and the GSE10927 dataset; **(E)** Time-dependent ROC analysis of the gene signature in pooled data from the GSE19750 dataset and the GSE10927 dataset; **(F)** Heatmap of the expression profiles of 5 prognostic genes in low-risk and high-risk group in pooled data from the GSE19750 dataset and the GSE10927 dataset. ACC, adrenocortical carcinoma; TCGA, The Cancer Genome Atlas; OS: overall survival.

Univariate Cox regression analysis showed that the risk score calculated based on our predictive model was significantly associated with poor prognosis in patients with ACC (*P* < 0.001, HR: 3.57, 95% CI: 2.39–5.32; Figure 7A). Multivariate Cox regression analysis suggested that the risk score was an independent risk factor for poor prognosis in patients with ACC (*p* < 0.001, HR: 4.21, 95% CI: 2.45–7.24; Figure 7B). A nomogram was established to predict the OS of ACC patients. Age, sex, stage, and the risk score were used to construct the nomogram. The risk score contributed the most to the prediction of OS (Figure 7C). To verify the generalizability of the predictive model, its predictive performance was externally validated using pooled data from the GSE19750 and GSE10927 datasets. Consistent with the results of the TCGA dataset, the risk score was significantly associated with OS in these datasets (*P* < 0.001; Figure 7D). ROC curve analysis confirmed the reliability and stability of the predictive model for predicting OS (AUC = 0.827; Figure 7E). The heatmap displays the difference in the expression profiles of the five prognostic genes in the low-risk and high-risk groups (Figure 7F).

### Functional Enrichment Analysis

**Figure 8:**
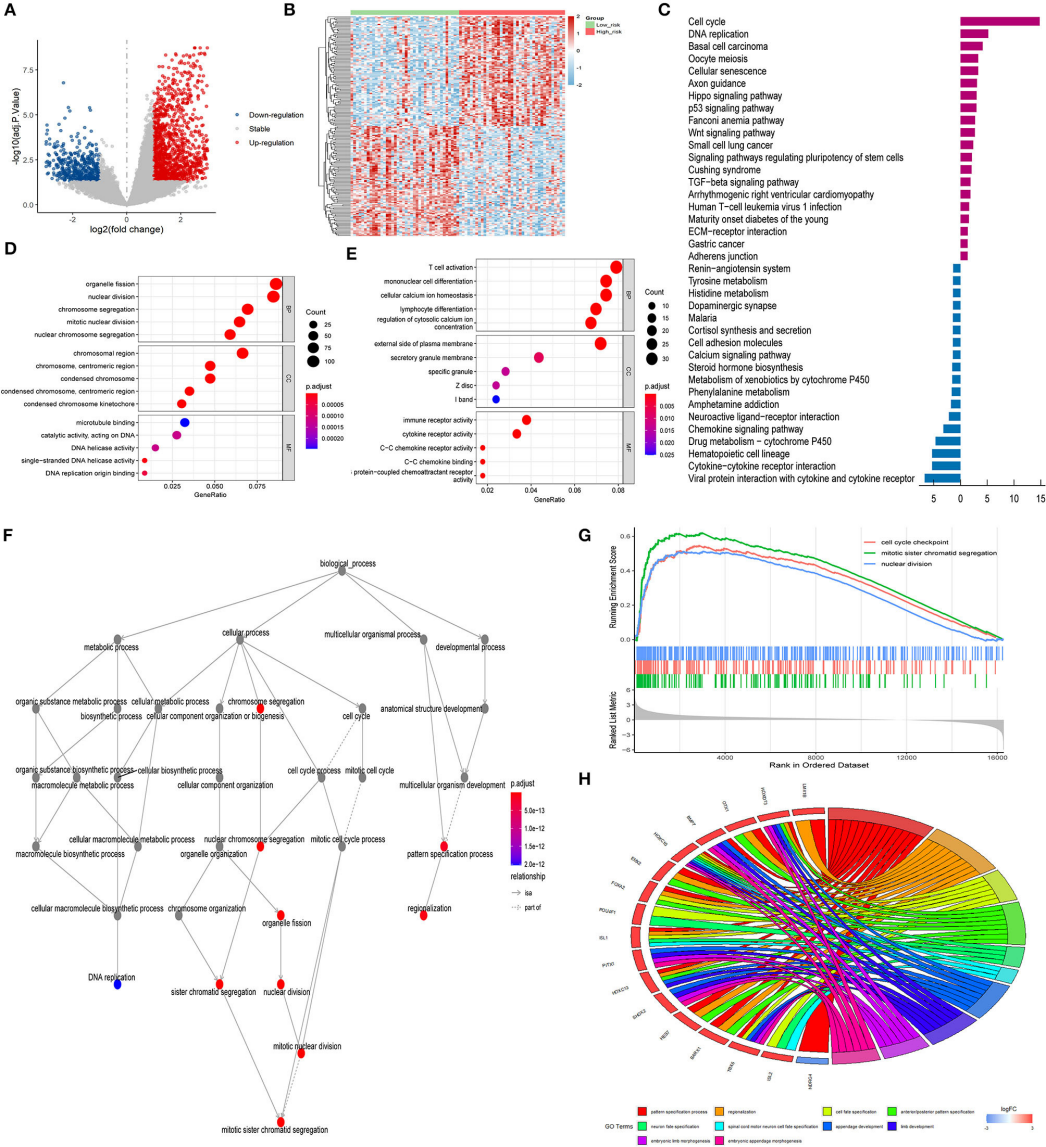
GO and KEGG analysis. **(A)** Volcano plots indicating up-expressed and down-expressed genes between different risk groups in ACC; **(B)** Hierarchical clustering analysis of genes between different risk groups in ACC; **(C)** KEGG analysis of DEGs between different risk groups in ACC (dark purple represented up-regulation; blue represented down-regulation); **(D)** GO analysis of up-regulated DEGs between different risk groups in ACC; **(E)** GO analysis of down-regulated DEGs between different risk groups in ACC; **(F)** Interactive relationship between enriched BP pathways; **(G)** GSVA enrichment analysis; **(H)** Chord diagram of GO biological terms for DEGs with the top 100 largest fold change. ACC, adrenocortical carcinoma; GO, Gene Ontology; KEGG, Kyoto Encyclopedia of Genes and Genomes; DEG, differentially expressed gene; BP, biological process; MF, molecular function; CC, cellular component; GSVA, Gene Set Variation Analysis.

To explore the biological functions and pathways that were related to the risk score, 1,860 DEGs (1,379 up-regulated genes and 481 down-regulated genes) between the high- and low-risk groups were obtained (Figures 8A,B). GO enrichment and KEGG pathway analyses were performed. Figure 8C displayed the significantly enriched KEGG pathways, including cell cycle, DNA replication, basal cell carcinoma, oocyte meiosis, and cellular senescence for up-regulated DEGs, and chemokine signaling pathway, drug metabolism-cytochrome P450, hematopoietic cell lineage, cytokine-cytokine receptor interaction, and viral protein interaction with cytokine and cytokine receptor for down-regulated DEGs. Figure 8D displayed the significantly enriched GO items in up-regulated DEGs, including organelle fission, nuclear division, and chromosome segregation for biological process (BP), and chromosomal region, chromosome, centromeric region, and condensed chromosome for cellular component (CC), and microtubule binding, catalytic activity, and DNA helicase activity for molecular function (MF). Figure 8E displayed the significantly enriched GO items in down-regulated DEGs, including T cell activation, mononuclear cell differentiation, and cellular calcium ion homeostasis for BP, and external side of plasma membrane, secretory granule membrane, and specific granule for CC, and immune receptor activity, cytokine receptor activity, and C-C chemokine receptor activity for MF. Figure 8F represented the interactive relationship between enriched BP pathways. GSVA enrichment analysis strongly implicated cell cycle checkpoint, mitotic sister chromatic segregation, and nuclear division as the main biological process (Figure 8G). The chord diagram in Figure 8H displayed GO biological terms for the top 100 genes with the largest fold change. GO terms including pattern specification process, regionalization, and cell fate specification were enriched.

### Pan-Cancer Analysis of Five Genes in the Predictive Model

**Figure 9:**
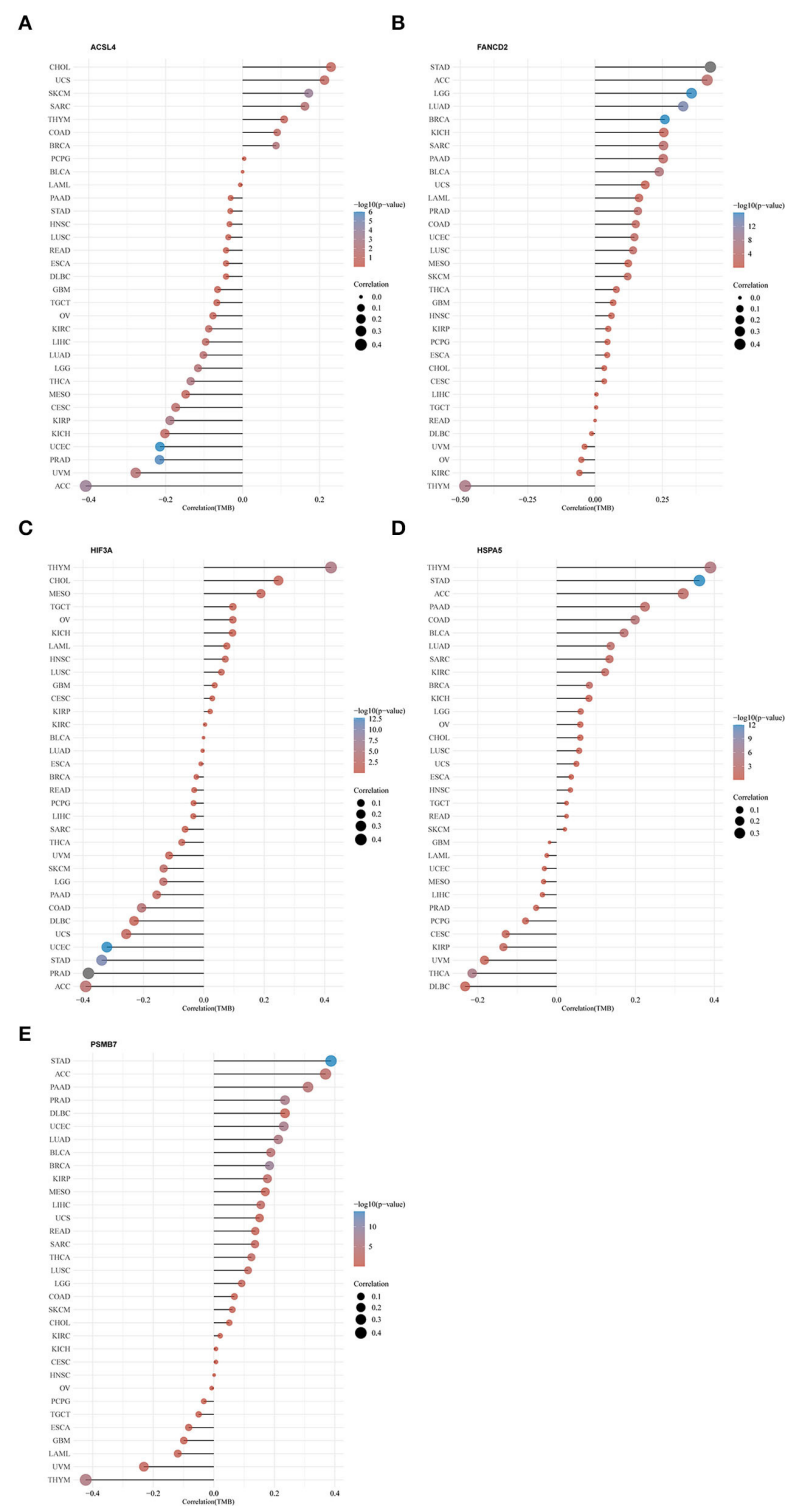
Pan-cancer analysis of five hub genes in the predictive model and TMB. **(A)** ACSL4; **(B)** FANCD2; **(C)** HIF3A; **(D)** HSPA5; **(E)** PSMB7. TMB: tumor mutation burden. The full names of the 33 cancers are available on the Cancer Genome Atlas (TCGA) database (https://portal.gdc.cancer.gov/repository).

We further explored the effect of 5 hub genes (ACSL4, FANCD2, HIF3A, HSPA5, and PSMB7) in 33 kinds of cancer in the TCGA database (Figure 9). Expressions of five genes were all significantly correlated with TMB in ACC. Among the 33 cancer types, ACSL4 expression was most correlated with TMB in ACC, whereas FANCD2, HIF3A, HSPA5, and PSMB7 were most correlated with TMB in thymoma (THYM).

## Discussion

ACC is a rare malignancy of the adrenal cortex. Despite its severe morbidity due to endocrine disturbances and tumor growth, there has been no remarkable progress in the treatment of ACC since the introduction of surgery and mitotane plus platinum-based therapy (1, 2, 22). The survival of patients with ACC has not improved substantially since 1993 (23). Further studies investigating the mechanisms that account for the poor prognosis of ACC and identifying more sensitive and effective biomarkers for early diagnosis, treatment, and prognosis of ACC are needed.

In this study, three hypoxia-related molecular subtypes (C1, C2, and C3) of ACC were identified based on the expression profiles of hypoxia-related genes. Our data demonstrated that patients with the C3 subtype had the shortest survival. Furthermore, the expression profile of ferroptosis-related genes differed significantly among the three hypoxia-related molecular subtypes. We found that ferroptosis-related genes, including ACSL4, ATL1, ATP5MC3, CARS1, CISD1, CS, DPP4, FANCD2, FDFT1, HSPA5, HSPB1, LPCAT3, NCOA4, NFE2L2, SLC1A5, SLC7A11, and TFRC, had distinct expression profiles in the three hypoxia-related ACC subtypes, indicating that HIF-1 signaling may contribute to dysfunction of ferroptosis in ACC. Intratumoral hypoxia is common in human cancer and has been found to increase the activity of HIFs that regulate angiogenesis, metabolic reprogramming, extracellular matrix remodeling, epithelial-mesenchymal transition, motility, invasion, metastasis, cancer stem cell maintenance, immune evasion, and resistance to chemotherapy and radiotherapy (24). Nonetheless, there are currently no perfect therapeutic drugs for clinical use that specifically target hypoxic cancer cells (25, 26). Many studies have reported that ferroptosis may play an important role in cancer progression (13, 14, 27). It has been found that ACC was remarkably sensitive to ferroptosis, indicating that the induction of ferroptosis may be a very promising treatment for ACC (18, 28). Mechanisms underlying susceptibility and resistance to ferroptosis remain unclear and the roles of HIF and ferroptosis in cancer progression have long received a lot of attention. Previous basic research found that Mobilization of lipids from droplets by HIF-1 signaling could lead to catabolism of polyunsaturated fatty acids and decrease their incorporation into phospholipids, thereby decreasing cellular sensitivity to ferroptosis (29). Our analysis of the profile of ferroptosis-related genes among three hypoxia-related molecular subtypes in patients with ACC also further confirmed the close relationship between HIF and ferroptosis in cancer progression.

Although there is much interest in the roles of HIF-1 and ferroptosis in the prognosis of cancer patients, no predictive model combining hypoxia- and ferroptosis-related gene expression has been created in ACC, probably because of the extreme rarity of ACC. In our study, we comprehensively studied the differential expression of genes related to hypoxia and ferroptosis in ACC and control samples. In addition, a novel prediction model integrating two hypoxia-related DEGs (HIF3A and PSMB7) and three ferroptosis-related DEGs (ACSL4, FANCD2, and HSPA5) was constructed and validated. It may be because the roles of hypoxia in the progression of ACC have not received enough attention, and because of the rarity of ACC, the role of HIF3A and PSMB7 in ACC has not been studied. However, considerable researches have been done in other cancers. HIFs are heterodimeric complex proteins that consist of an alpha subunit (HIF-1α, HIF-2α, or HIF-3α) and a beta subunit (7). HIF-1 governs the acute adaptation to hypoxia, whereas HIF-2 and HIF-3 expressions begin during chronic hypoxia in human endothelium. When HIF-1 levels decline, HIF-2 and HIF-3 increase (30). A few studies have shown that the gene expression of HIF3A was downregulated in breast cancer and non-small cell lung cancer (31, 32). Silakit et al. found that HIF-3α sustained HIF-1α activity and regulated cell growth and chemotherapeutic drug resistance in cholangiocarcinoma cells (33). Maynard et al. found that HIF-3α4, an alternatively spliced variant of human HIF-3α, prevented the engagement of HIF-2 to the hypoxia-responsive elements located in the promoter/ enhancer regions of hypoxia-inducible genes, thus suppressed the tumor growth of VHL-null renal cell carcinoma (34). Proteasomes are involved in vital processes including cell cycle regulation, apoptosis, and angiogenesis; therefore, they represent an attractive target for anticancer therapy (35). PSMB7 encodes the β-type proteasome subunit 7. Rho et al. found the overexpression of PSBM7 in colon adenocarcinoma and the specific location of PSBM7 up-regulation within heterogeneous primary human tumor tissue was confirmed by immunohistochemistry (36). Munkacsy et al. found that PSMB7 was associated with anthracycline resistance and the patients with high PSMB7 expression had significantly shorter survival than the patients with low expression in breast cancer (37). Tan et al. found that PSMB7 was associated with 5-fluorouracil resistance in hepatocellular carcinoma. Furthermore, down-regulation of PSMB7 enhanced hepatocellular carcinoma to 5-fluorouracil sensitivity (38). Ferroptosis, a recently identified form of non-apoptotic cell death, is involved in cancer progression (39). ACSL4, an essential regulator of lipid metabolism, is identified as a biomarker and contributor to ferroptosis (40). ACSL4 enriched cellular membranes with long polyunsaturated ω6 fatty acids. Moreover, ACSL4 was found preferentially expressed in a panel of basal-like breast cancer cell lines and predicted their sensitivity to ferroptosis (41). A ferroptosis signature comprised of six genes including ACSL4 was found to be associated with prognosis and immune infiltration in ACC (18). In other cancers, Sha et al. found that higher ACSL4 expression was related to better overall survival in breast cancer (42). Cheng et al. found that ACSL4 suppresses glioma cell proliferation by activating ferroptosis (43). Luo et al. found that higher ACSL4 expression was associated with CD8+ T cell infiltration and immune response in bladder cancer (44). FANCD2 is a nuclear protein involved in DNA damage repair and has been found to protect against ferroptosis (45). Wu et al. found that a new ferroptosis signature including FANCD2 accurately predicted prognosis in clear cell renal cell carcinoma (46). Fagerholm et al. found that overabundant FANCD2 was a sensitive marker of adverse prognosis in breast cancer (47). Moes-Sosnowska et al. found that FANCD2 overexpression was a strong negative prognostic factor in ovarian cancer, particularly in patients treated with taxane-platinum regimen (48). HSPA5 is a molecular chaperone expressed primarily in the endoplasmic reticulum and is closely associated with tumor progression and poor prognosis (49). In ACC, Ruggiero et al. found that the HSPA5 inhibitor HA15 synergized with mitotane action against adrenocortical carcinoma cells through convergent activation of endoplasmic reticulum stress pathways (50). Growing evidence showed that HSPA5 mediated ferroptosis resistance and negatively regulated ferroptosis in cancer cells (51). Our study suggests that these genes are important prognostic factors in ACC and could be potential therapeutic targets, although the roles of these hub genes in ACC still need to be further investigated.

We further perform bioinformatic enrichment analysis to explore the biological functions and pathways that were related to the risk score calculated by the predictive model. The significantly enriched KEGG pathways included cell cycle, DNA replication, basal cell carcinoma, oocyte meiosis, and cellular senescence for up-regulated DEGs, and chemokine signaling pathway, neuroactive ligand-receptor interaction, amphetamine addiction, phenylalanine metabolism, and metabolism of xenobiotics by cytochrome P450 for down-regulated DEGs. We also found that the expressions of five hub genes were all significantly associated with TMB in ACC, suggesting that the predictive model has the potential to be used for predicting response to immune checkpoint inhibitor therapy. However, further studies will be necessary to investigate the underlying mechanisms.

Our study has several limitations. First, our analysis shared the limitations of the TCGA and GEO datasets. The accuracy of the TCGA and GEO datasets is limited by the quality and availability of the original data. Nevertheless, therapeutic targets including Fibroblast Growth Factor 19 (FGF19), have been successfully validated in pre-clinical settings after they were identified from bioinformatic analysis (52), which motivates us to continue bioinformatic analysis of some refractory tumors, including ACC. Second, this is a retrospective study. The predictive model should be validated in a large, multicenter ACC cohort. Third, further functional and mechanistic studies are required to elucidate hypoxia-ferroptosis interactions and the underlying cancer pathogenesis. Notwithstanding these limitations, the findings of this study could yield novel therapeutic targets for ACC.

## Conclusion

In conclusion, this study identified three hypoxia-related molecular subtypes with distinct prognoses and ferroptosis-related gene expression profiles in ACC patients. A predictive model combining hypoxia- and ferroptosis-related gene expression was constructed and validated. This model could accurately and powerfully predict the prognosis of patients with ACC. We also established a nomogram combining age, sex, tumor stage, and our predictive model to assist in clinical judgment. These findings provide new ideas for the diagnosis, prognostic prediction, and treatment of ACC.

## Data Availability Statement

The datasets presented in this study can be found in online repositories. The names of the repository/repositories and accession number(s) can be found in the article/Supplementary Material.

## Author Contributions

All authors were involved in drafting the manuscript, and have read and approved the final version.

## Funding

This work was supported by grants from the National Natural Science Foundation of China (81870564 and 81670744).

## Conflict of Interest

The authors declare that the research was conducted in the absence of any commercial or financial relationships that could be construed as a potential conflict of interest.

## Publisher's Note

All claims expressed in this article are solely those of the authors and do not necessarily represent those of their affiliated organizations, or those of the publisher, the editors and the reviewers. Any product that may be evaluated in this article, or claim that may be made by its manufacturer, is not guaranteed or endorsed by the publisher.
