# Supplementary material for: A Novel Predictive Model for Adrenocortical Carcinoma Based on Hypoxia- and Ferroptosis-Related Gene Expression
Source: Front Med (Lausanne). 2022 May 16;9:856606. doi: 10.3389/fmed.2022.856606 (PMC9148996; doi:10.3389/fmed.2022.856606)
Supplement: Supplementary file 1 [file Data_Sheet_1.docx]

Supplementary Material

# 1 Supplementary Tables

**Supplementary Table 1.** The list of hypoxia-related genes.

| **75 hypoxia-related genes** | | | | |
| --- | --- | --- | --- | --- |
| PSMB6 | UBC | UBE2D1 | PSMB8 | SEM1 |
| PSMB5 | PSMD11 | PSMD14 | PSMA4 | EPAS1 |
| HIGD1A | PSMD10 | PSMC4 | VHL | PSMA5 |
| EGLN2 | PSMB10 | PSMB2 | HIF3A | PSMA2 |
| PSMD1 | PSMD5 | PSMB4 | WTIP | EPO |
| PSMA7 | ELOB | LIMD1 | EGLN3 | PSME3 |
| HIF1AN | PSME2 | PSMD2 | ARNT | PSMD7 |
| PSMC2 | CREBBP | PSMB7 | PSMD12 | PSMB9 |
| PSMD3 | UBB | PSMA1 | PSMA6 | PSME1 |
| EP300 | PSMD6 | PSMA8 | EGLN1 | HIF1A |
| VEGFA | PSMD13 | PSMC6 | PSMB3 | CITED2 |
| ELOC | PSMB11 | PSMD9 | PSMD8 | UBA52 |
| PSMC3 | CA9 | RBX1 | CUL2 | UBE2D3 |
| PSME4 | PSMF1 | PSMC5 | PSMA3 | PSMD4 |
| UBE2D2 | AJUBA | PSMB1 | PSMC1 | RPS27A |

**Supplementary Table 2.** The list of ferroptosis-related genes.

| **24 ferroptosis-related genes** | | | | |
| --- | --- | --- | --- | --- |
| LPCAT3 | GLS2 | RPL8 | ATP5MC3 | SLC7A11 |
| HSPA5 | ALOX15 | FANCD2 | GPX4 | HSPB1 |
| CARS | SAT1 | NFE2L2 | FDFT1 | CISD1 |
| CDKN1A | ACSL4 | DPP4 | MT1G | SLC1A5 |
| CS | EMC2 | TFRC | NCOA4 |  |
